# Characterization of the Fat Channel for Intra-Body Communication at R-Band Frequencies

**DOI:** 10.3390/s18092752

**Published:** 2018-08-21

**Authors:** Noor Badariah Asan, Emadeldeen Hassan, Jacob Velander, Syaiful Redzwan Mohd Shah, Daniel Noreland, Taco J. Blokhuis, Eddie Wadbro, Martin Berggren, Thiemo Voigt, Robin Augustine

**Affiliations:** 1 Microwaves in Medical Engineering Group, Solid State Electronics, Department of Engineering Sciences, Ångström Laboratory, Uppsala University, P.O. Box 534, 751 21 Uppsala, Sweden; jacob.velander@angstrom.uu.se (J.V.); syaiful.redzwan@angstrom.uu.se (S.R.M.S.); 2 Faculty of Electronic and Computer Engineering, Universiti Teknikal Malaysia Melaka, Durian Tunggal 76100, Malaysia; 3 Department of Computing Science, Umeå University, 901 87 Umeå, Sweden; emad@cs.umu.se (E.H.); noreland@cs.umu.se (D.N.); eddiew@cs.umu.se (E.W.); martinb@cs.umu.se (M.B.); 4 Department of Electronics and Electrical Communications, Menoufia University, Menouf 32952, Egypt; 5 Department of Surgery, Maastricht University Medical Center, 6229 HX Maastricht, The Netherlands; taco.blokhuis@mumc.nl; 6 Department of Information Technology, Uppsala University, 752 36 Uppsala, Sweden; thiemo@sics.se

**Keywords:** intra-body communication, path loss, microwave probes, channel characterization, fat tissue, ex-vivo, phantom, dielectric properties, topology optimization

## Abstract

In this paper, we investigate the use of fat tissue as a communication channel between in-body, implanted devices at R-band frequencies (1.7–2.6 GHz). The proposed fat channel is based on an anatomical model of the human body. We propose a novel probe that is optimized to efficiently radiate the R-band frequencies into the fat tissue. We use our probe to evaluate the path loss of the fat channel by studying the channel transmission coefficient over the R-band frequencies. We conduct extensive simulation studies and validate our results by experimentation on phantom and ex-vivo porcine tissue, with good agreement between simulations and experiments. We demonstrate a performance comparison between the fat channel and similar waveguide structures. Our characterization of the fat channel reveals propagation path loss of ∼0.7 dB and ∼1.9 dB per cm for phantom and ex-vivo porcine tissue, respectively. These results demonstrate that fat tissue can be used as a communication channel for high data rate intra-body networks.

## 1. Introduction

Data from the World Health Organization indicates that millions of people are suffering from noncommunicable diseases (NCDs), such as cardiovascular conditions, chronic respiratory diseases, cancer, diabetes, obesity, or arthritis [1,2]. In response to the immense societal costs associated with such conditions, great efforts are made to develop various medical devices for diagnosis, treatment, or real-time monitoring [3]. Many such devices are implanted into the body. Examples are cardiac pacemakers, cochlear implants, defibrillators, nerve and diaphragm stimulators, active drug administration devices, and implantable active monitoring devices [4,5]. The devices often need to communicate with the outside world or with other implants elsewhere in the body. For reasons related to infection concerns, mobility, and accessibility, wireless interfaces are often preferred to wired techniques. In light of this, intra-body communication has been considered for many medical applications over the past few years and still attracts increasing attention [6,7]. Referred to as Intra-body Area Networks (i-BANs), the communication links encompass on-body, off-body, and implanted (in-body) nodes.

Moreover, wireless body-centric sensing systems also have an important role in the fields of biomedicine, personal healthcare, safety, and security [8]. Simultaneous aggregation of information from multiple implanted devices and stimulators is needed for the therapist to administer treatments. This will increase the demand on the data capacity of the communication channel.

Previous investigators have employed three different physical principles for intra-body communication: galvanic coupling [9,10,11], capacitive coupling [12,13,14], and Radio Frequency (RF) links [15,16]. The Medical Implant Communication Service (MICS) operating over the frequency range 402–405 MHz has been accepted worldwide for data transmission to support medical application associated with medical implant devices. This frequency range limits the transfer of image data because of the bandwidth and regulatory limitations [17,18]. A solution to this could be found in modern wireless technology and it would play an essential role in making telemedicine possible.

The microwave intra-body communication through the fat tissue, coined as a “fat channel” [19], is a new technique that uses the fat tissue as a communication channel. Previous studies have proven the feasibility of using the fat tissue as a transmission medium for microwave signals at 2.0 GHz [19,20]. There is still a need to improve the characterization of the microwave communication through the fat tissue, which has not been taken into account in previous research.

Asan et al. [20] have successfully shown approximately 96% data packet reception through the fat tissue for a fat channel of length 10 cm. It has been shown that the communication through the fat tissue is still possible even at 60% obstruction due to embedded muscle tissues [21]. To design stable and reliable fat iBAN links, it is important to assess the microwave propagation losses along the fat channel. This will allow the development of reliable in-body communication systems for real-time and continuous monitoring.

The focus of this paper is to investigate the propagation of microwave signals through the fat tissue and scrutinize the performance of the intra-body communication technique with an ambition to gather data from multiple implanted devices or stimulators in the human body by utilizing the fat tissue. To study the properties of the fat channel, we need to design probes that can launch/receive signals to/from the fat tissue.

In this paper, for the first time, we present a novel probe that is matched to the fat tissue over the whole R-band. We optimize our probe by using a computational topology optimization approach [22]. The optimized probe is used to characterize the sender–fat-channel–receiver link and compare its performance to other communication channels. In particular, we compare the signal transmission through the fat channel to the signal transmission in free space, between parallel plate waveguides, and through a dielectric waveguide formed by the fat tissue and the surrounding skin and muscle tissue. We use numerical simulations and experimental measurements to conduct our investigations. We perform experimentations using a phantom and an ex-vivo porcine tissue to validate our results.

In the following section, we briefly describe the fat channel concept. In Section 3, we present our optimized probe and its performance validation. The characterization of the fat channel over the R-band frequencies is described in Section 4. Finally, the conclusions and some finding remarks are summarized.

## 2. Fat Channel Model

**Figure 1:**
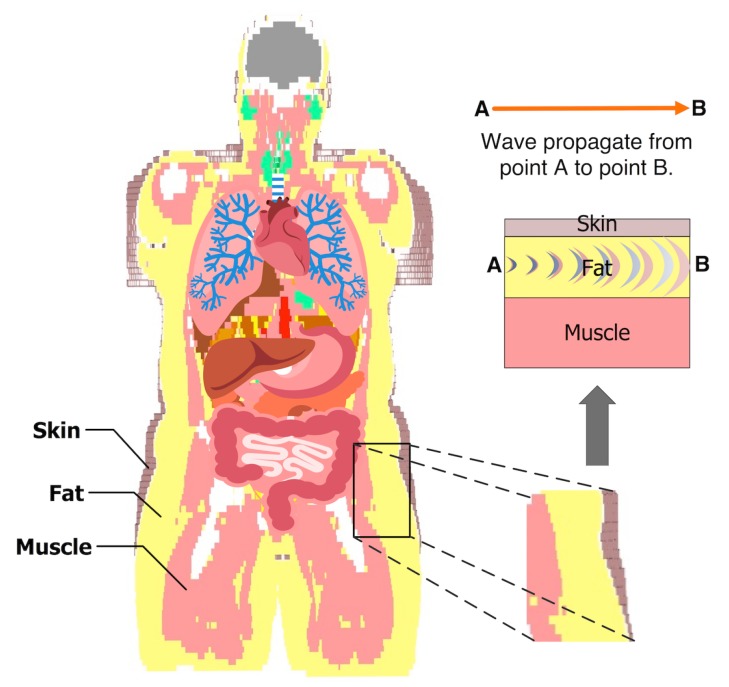
The model shows the fat tissue surrounding the vital organs in the abdomen. The example of the three tissues is taken to characterize the intra-body microwave transmission through the fat tissue.

Figure 1 shows a coronal plane of the Emma voxel human model from the Computer Simulation Technology (CST) software [23]. The image shows that fat tissue is omnipresent and surrounds the important organs in the human body. The fat tissue has low losses compared to those of the skin and the muscle tissues. This property makes the fat tissue a suitable choice for communication between implanted devices. The distribution of the skin, fat, and muscle tissues, shown in Figure 1, has been the motivation for our sample model selection.

**Figure 2:**
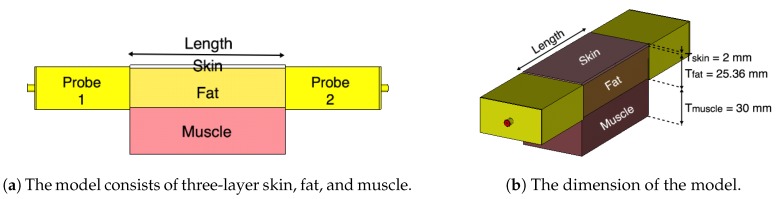
The three-layered tissue model structure.

Figure 2a shows a cross section of our generic model of the fat channel. The model consists of three layers, skin, fat, and muscle, in order to exemplify human tissue structure. The thickness of the skin, fat, and muscle are 2 mm, 25.36 mm, and 30 mm, respectively. These dimensions are chosen based on the average human body tissue thickness and in accordance with previous studies [19,20,24]. The 2 mm thick skin is chosen by taking the average thickness of human adult [25]. The average thickness of human fat tissue is found to be 25 mm [26]. The 30 mm muscle thickness is chosen for the current study based on our previous numerical investigations [19].

Our numerical investigations showed little impact on increasing the channel width on the characterization of the fat channel. Thus, in all of our investigations, we choose a channel width of 50.72 mm for the three tissues, similar to the size of the probe’s aperture used in our study. In the simulations and the experiments, we vary the channel length between 20 mm and 100 mm, in 20 mm steps, to obtain a relative measure of the channel path loss. Figure 2b shows the three-layer tissue model with two probes attached to the opposite sides of the channel.

**Figure 3:**
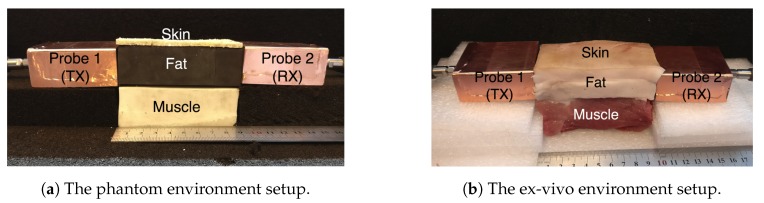
The three-layer fat channel that consists of the skin, fat, and muscle tissues arranged in order from top to bottom.

We use the CST Microwave Studio package (version 2018.06) to numerically simulate the proposed model. We carry out experimentations by using phantom and ex-vivo porcine tissue. Figure 3 shows the three-layer phantom-equivalent and ex-vivo porcine belly tissue measurement setup.

The phantoms are fabricated by using agar-based compounds that represent the skin and the muscle tissue, and a solid rubber block that represents the fat tissue. We have previously used adhesive putty as a fat equivalent phantom [19]. In this study, we choose solid rubber over adhesive putty due to its solid nature, and since it can be easily machined into different geometrical shapes.

The ex-vivo experiment uses a three-layer porcine belly tissue that is obtained from a local slaughterhouse. The dielectric properties of the fabricated phantom and the ex-vivo porcine tissue are measured with an Agilent 85070E Dielectric Probe Kit. The simulation models are assigned with the respective dielectric properties of the constituent materials measured during the experiments to closely compare the path loss between the simulation and the measurement. The dielectric properties of the phantom, the ex-vivo, and human tissues at 2.4 GHz are presented in Table 1, for skin, fat, as well as muscle tissue. Similarly to what was done by Gabriel and Peyman [27], we report the Type B uncertainties expanded to a required confidence level of approximately 95% using a rectangular distribution . The Type A uncertainties were too low when compared to the Type B ones and, therefore, are neglected. For the phantom and ex-vivo models, the Type B uncertainty was estimated using the accuracy reported in the datasheet of the Agilent Kit, which was ∼10% of the mean. For the human model, the uncertainty discussion in [28] suggests that we can use the same accuracy and uncertainty model.

To assess the fat channel for signal transmission, we need to launch signals into the fat tissue. In the next section, we present a new microwave probe optimized to launch microwave signals at the R-band frequencies into the fat tissue.

## 3. Probe Design Optimization

In this section, we propose a new microwave probe optimized to transmit R-band frequencies into fat tissue. The probe design is carried out by using a state-of-the-art numerical optimization technique known as topology optimization. We optimize the probe in a setup similar to where it will be used. In particular, the probe is optimized in an environment suitable for intra-body communication through the fat tissue.

### 3.1. Probe Optimization

**Figure 4:**
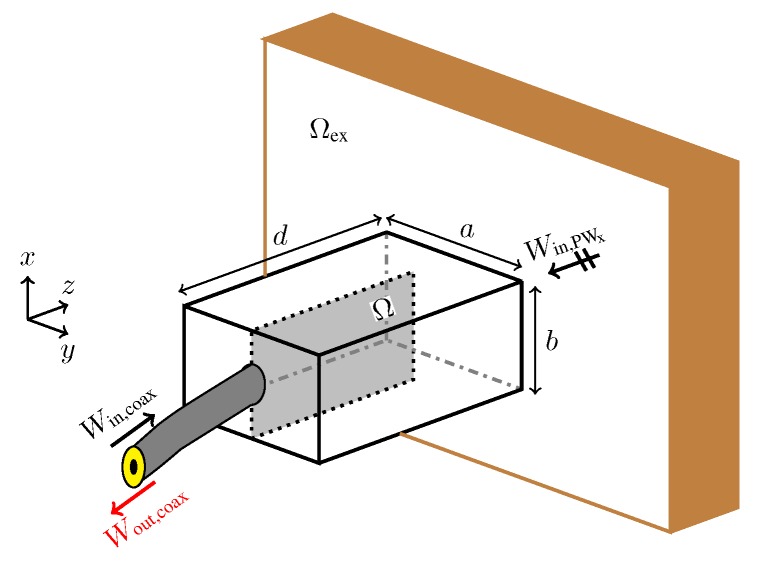
The setup for the probe optimization. The objective is to find the conductivity distribution in Ω that maximizes the energy coupling between the probe and the exterior domain $\Omega_{\mathrm{ex}}$ (the fat tissue).

Figure 4 shows the setup used for the probe optimization. The probe consists of a rectangular waveguide section placed between a coaxial cable and the fat tissue. The waveguide section has dimensions a=50.72 mm, b=25.36 mm, and d=60.59 mm (see Figure 4). To maximize the signal coupling over the frequency band 1.3–2.9 GHz, we aim to distribute a metallic material (copper) in the design domain Ω located on one side of a printed circuit board. The domain Ω has dimensions 25.36×50.72 mm${}^{2}$ and is backed by a Rogers RO-3203 substrate with ϵ=3.02 to hold the copper distribution. Note that the presence of the waveguide section prevents interference of the probe with the outside environment. Moreover, it is possible to use standard printed circuit board processes to fabricate the copper distribution in Ω, which facilitates the probe manufacturing and assembly.

During the probe optimization, the exterior domain $\Omega_{\mathrm{ex}}$ is modelled to fill half the space with a material possessing the same dielectric properties as those of the phantom fat tissue in Table 1. To decrease the probe size and to improve the matching, we fill the interior of the waveguide section with the same dielectric material as the domain $\Omega_{\mathrm{ex}}$.

To design the waveguide probe, we formulate the optimization problem

(1) $$\begin{array}{cc} \max_{\sigma_{\Omega }}\; & \log\left(\frac{W_{\mathrm{out},\mathrm{coax}}{|}_{W_{\mathrm{PWx}}}}{W_{\mathrm{out},\mathrm{coax}}{|}_{W_{\mathrm{in},\mathrm{coax}}}}\right),\\ s.t.\; & \mathrm{the}\;\mathrm{governing}\;\mathrm{equations}\;\mathrm{and}\;\mathrm{excitation}\;\mathrm{signals}, \end{array}$$

where $W_{\mathrm{out},\mathrm{coax}}{|}_{W_{\mathrm{PWx}}}$ is the outgoing energy in the coaxial cable when an *x*-polarized plane wave propagates from $\Omega_{\mathrm{ex}}$ towards the probe, $W_{\mathrm{out},\mathrm{coax}}{|}_{W_{\mathrm{in},\mathrm{coax}}}$ is the reflected energy to the coaxial cable when the signal source is the coaxial cable. Problem (1) seeks the conductivity distribution $\sigma_{\Omega }$ that matches the signal from the coaxial cable to $\Omega_{\mathrm{ex}}$ by maximizing the transmission term in the numerator and minimizing the reflection term in the denominator.

To solve optimization Problem (1), we use the material distribution approach to topology optimization [29]. This approach has recently been used for optimizing various electromagnetic components such as antennas, filters, and waveguide transitions [30,31,32,33]. In this approach, the design domain is discretized into small pixels and a design variable is assigned to each pixel to indicate presence or absence of metal. In the material distribution approach, design variables are allowed to vary continuously between two limits during the optimization. This enables the use of gradient-based optimization methods to solve such problems. By the end of the optimization, design variables typically have binary values (metal or dielectric) enforced using different numerical techniques.

To evaluate the objective function in Problem (1), we numerically solve Maxwell’s equations using the finite-difference time-domain (FDTD) method [34]. We use a uniform spatial step of 0.2818 mm and a temporal step of 0.9 times the Courant limit. We discretize the design domain Ω into 90×180 cells, which results in 32,220 design variables, associated with the interior edges of the computational grid. To compute the two terms in the objective function, we use two FDTD simulations. In one simulation, we excite the problem with a plane wave from the tissue side and evaluate $W_{\mathrm{out},\mathrm{coax}}{|}_{W_{\mathrm{PWx}}}$. That is, in this case, the probe is operating in receiving mode. In the second simulation, the problem is excited through the coaxial cable and we evaluate the reflection term $W_{\mathrm{out},\mathrm{coax}}{|}_{W_{\mathrm{in},\mathrm{coax}}}$. The use of the reflection term in the objective function formulation typically results in designs with better performance compared to when only the transmission term is used. In both cases, we use a time-domain *sinc* signal with frequency content that cover the frequency band 1.3–2.9 GHz.

Any gradient-based optimization method used to solve Problem (1) requires the objective function gradient. We employ the adjoint field method and derive an adjoint system based on the FDTD discretization of Maxwell’s equations [33]. We combine the adjoint system solution and the forward field solution to compute the objective function gradient in an efficient manner. More precisely, for any number of design variables, we compute the objective function gradient on the basis of four system solutions: two solutions for the objective function evaluations, as described above, and two solutions for the corresponding adjoint systems. We iteratively solve Problem (1) by using the method of moving asymptotes [35].

**Figure 5:**
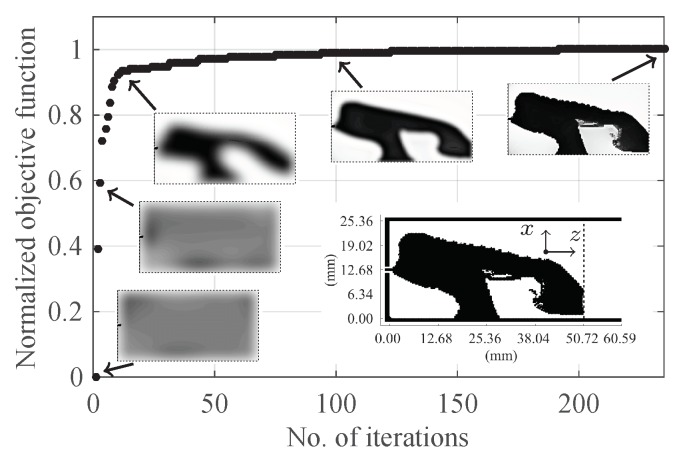
The progress of the objective function and some snapshots showing the development of the design. The final design is the insert inside the figure with the black colour indicating copper.

Figure 5 shows the progress of objective function and some snapshots that show the development of the design during the optimization process. The optimization problem starts with a uniform distribution of the electric conductivity ($\sigma_{i}∼2\times 10^{3}$ S/m) over the design domain. After 15 iterations, the solution of the optimization problem is a design with blurred boundaries (the third snapshot in the figure). The black colour is a good conductor and the white colour is a good dielectric. The optimization algorithm converges after 235 iterations. The final design has crisp boundaries and consists essentially of black and white colours. In a final post-processing step, we use a threshold conductivity $\sigma_{\mathrm{th}}=1$ S/m to map values above $\sigma_{\mathrm{th}}$ to $5.8\times 10^{7}$ S/m (copper) and values below $\sigma_{\mathrm{th}}$ to $9\times 10^{-3}$ S/m (the phantom’s conductivity). The final design is the insert in Figure 5.

**Figure 6:**
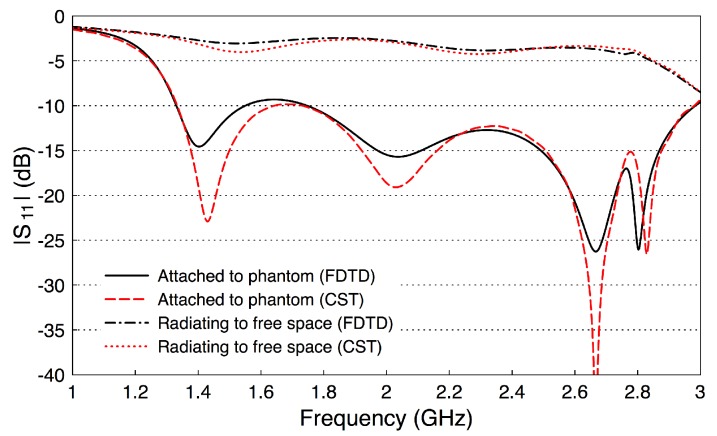
The reflection coefficient of the optimized probe when attached to the fat phantom and when radiating to free space.

To evaluate the performance of the optimized probe, we compute its reflection coefficient. (The probe-to-probe coupling will be evaluated in the next section.) Figure 6 shows the computed reflection coefficient ($|S_{11}|$ dB) for the probe when attached to the phantom and when the probe radiates in free space. The $|S_{11}|$ is computed with our FDTD code and cross-verified with the CST package with good match between the two methods. When the probe is attached to the phantom, $|S_{11}|$ is below −9.3 dB over the frequency band 1.33–2.97 GHz. However, $|S_{11}|$ is essentially above −4 dB when the probe radiates into free space. The probe has less reflection when attached to the phantom and the reflection coefficient deteriorates when it radiates into free space. This result shows that probes should be optimized in a setup similar to where they will be used.

### 3.2. Probe Fabrication and Validation

**Figure 7:**
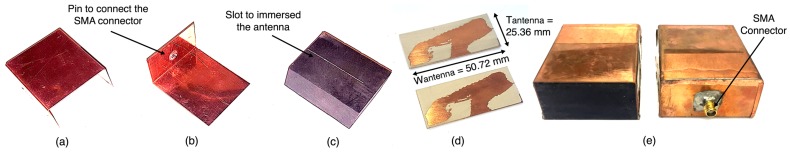
Probe assembly. (**a**) top covering copper plate; (**b**) bottom covering copper plate with an attached SMA connector; (**c**) fat-equivalent phantom with a slot to immerse the topology optimized antenna; (**d**) the topology-optimized antenna; (**e**) the final assembled probe.

Figure 7 shows the steps of the probe fabrication. The rectangular waveguide section is constructed of two bent copper plates (Figure 7a,b) and is filled with the fat-equivalent phantom, the solid rubber block in Figure 7c. A slot is cut at the center of the rubber block to immerse the topology optimized planar antenna (TOPA). Figure 7d is an annotated photograph of the TOPA fabricated on a Rogers RO-3203 substrate with epsilon ϵ = 3.02. The TOPA ($T_{antenna}$ = 25.36 mm and $W_{antenna}$ = 50.72 mm) is slid into the slot at the center of the rubber block, and a 50-Ω sub-miniature version A (SMA) connector is connected to the side of the TOPA. The components of the probe are assembled together, and the edges are soldered to form the final probe as illustrated in Figure 7e. In the characterization of the fat channel, we fabricated two prototypes of the probe, to use one as a transmitter and the other as a receiver, as described below.

**Figure 8:**
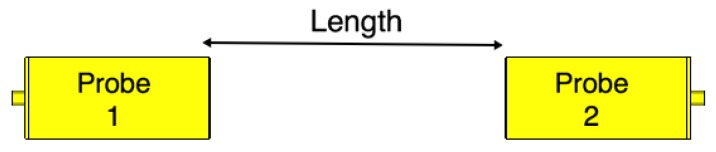
Probe-to-probe setup. The channel length is varied from 0 mm to 100 mm with 20 mm steps.

**Figure 9:**
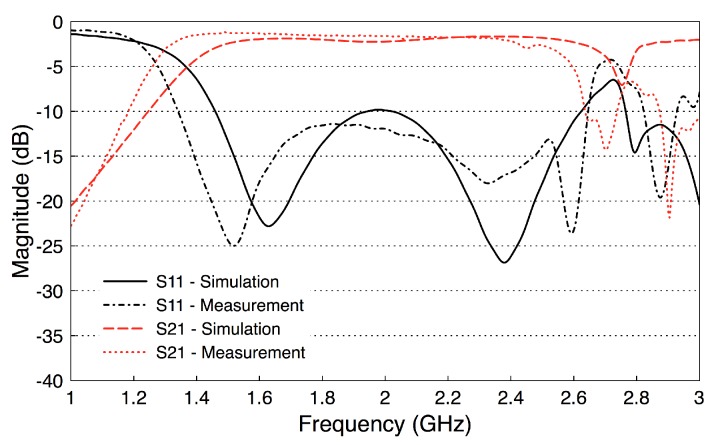
Measured and simulated S-parameters of the probe-to-probe setup (the channel length is zero).

To validate the performance of the fabricated probes, we measure their scattering parameters. Figure 8 illustrates a back-to-back setup of the probes. We set the channel length as a variable that will be used later in our investigations. We measure the scattering parameters with an Agilent Microwave FieldFox analyzer (N9918A). The measured data is acquired at 801 frequency points between 1 and 4 GHz. Figure 9 shows the measured and simulated scattering parameters of the two probes when the channel length is zero. There is a good match between the simulation and measurement results. The slight differences between the simulations and measurements can be attributed to fabrication errors and marginal errors in the measurements of the dielectric properties of the rubber block (the fat phantom).

The results show that $|S_{21}|$ has essentially a constant value of −2 dB over the frequency band 1.6–2.6 GHz, including the whole R-band frequencies and the Industrial, Scientific, and Medical (ISM) radio frequency band (2.4 GHz). In addition, the values of $|S_{11}|$ are smaller than −10 dB over the same frequency band. Note that these probe-to-probe results will be used as a reference for the subsequent measurements.

**Figure 10:**
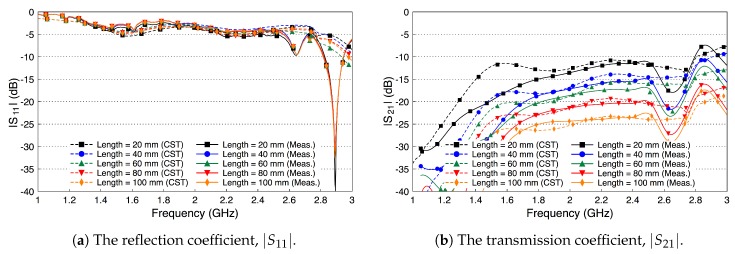
The scattering parameters of the probe-to-probe setup with a free space channel in between.

Similar to the single probe study, here we assess the scattering parameters of the probe-to-probe setup when the medium between the two probes is free space. Figure 10 shows the scattering parameters of the two probes when the channel length varies from 20 mm to 100 mm with a step size of 20 mm. We notice that the deterioration in the values of $|S_{11}|$ in Figure 10a is similar to the single probe case in Figure 6. In addition, there are minor variations in the $|S_{11}|$ values when the channel length between the two probes is varied. Figure 10b shows that the coupling coefficient $|S_{21}|$ decreases by almost 10 dB when the two probes are separated by a 20 mm free space channel. $|S_{21}|$ continues to decrease when increasing the free space channel length. On average, there is a decrease factor of 2 dB for each cm distance.

## 4. Fat Channel Characterization

### 4.1. Path Loss Characterization

In this section, we use our optimized probe to characterize the average path loss of the fat channel. We experiment both with the phantom and the ex-vivo porcine tissues. In addition, we compare our experiments in both cases with simulations by using the CST software package.

**Figure 11:**
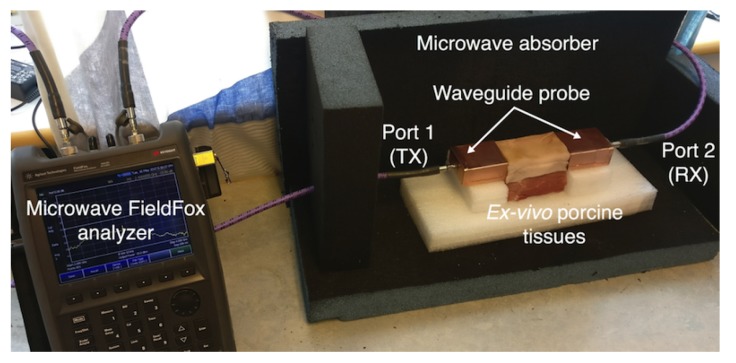
Experimental setup for three-layers ex-vivo porcine tissue.

Figure 11 shows the experimental setup for the three-layer ex-vivo porcine tissue. The waveguide probes are attached to the fat tissue on one side and connected through the SMA connector to the ports of the microwave network analyzer. The probes and the tissues are horizontally aligned. We surround the setup with microwave absorber foam to reduce the electromagnetic interference with the surroundings. We study the signal coupling through the fat channel for different channel lengths.

**Figure 12:**
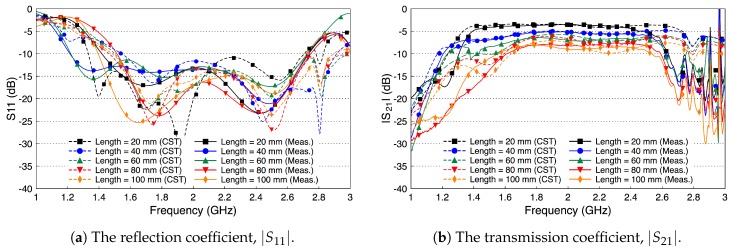
Measured and simulated scattering parameters of the three-layered tissue phantom.

Figure 12 shows the simulated and measured scattering parameters of the phantom experiment. The probe reflection coefficient $|S_{11}|$ is shown in Figure 12a for different channel lengths. We vary the channel length from 20 mm to 100 mm in steps of 20 mm. The amplitude of the $|S_{11}|$ curves are below −10 dB over the whole R-band frequencies, which indicates that the probe is well matched to the channel regardless of its length. There is a good match between the simulated and measured results. Figure 12b shows the coupling coefficient $|S_{21}|$ over the fat channel phantom for the different channel lengths. The amplitude of $|S_{21}|$ is almost flat over the R-band frequencies and varies from −3.5 dB to around −9 dB as the channel length varies from 20 mm to 100 mm, respectively. The average path loss is around 0.7 dB for each cm phantom channel length. Note that the phantom loss is quite small compared to the human or the ex-vivo tissues. Nevertheless, this phantom can be used to facilitate the investigations of different channel scenarios.

**Figure 13:**
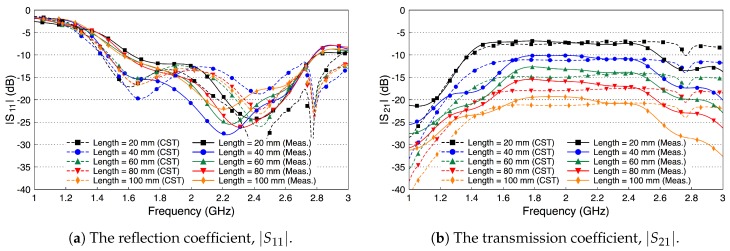
Measured and simulated scattering parameters of the three-layered tissue in ex-vivo porcine tissue.

Figure 13 shows the simulated and measured scattering parameters of the fat channel when the ex-vivo porcine tissue is used. The probe’s reflection coefficient, shown in Figure 13a, is smaller than −10 dB for all tested channel lengths. These small values of the reflection coefficient indicate that the probe launches most of the signal into the fat tissue. In general, there is a good agreement between the simulated and measured results. Figure 13b shows the coupling coefficient through the porcine tissue. The $|S_{21}|$ curves are essentially flat over the R-band frequencies. The amplitude of $|S_{21}|$ is −7 dB for the 20 mm channel length and decreases essentially by 1.9 dB for each cm increase in the channel length.

In view of the obtained results, the propagation losses of the phantom and the ex-vivo environments are ∼0.7 dB and ∼1.9 dB per cm, respectively. The new results of the phantom and ex-vivo are in agreement with our previous work [19,20]. Since human tissue properties, as reported in [28], are between the properties of the phantom and the ex-vivo porcine tissue used in this study, we expect the propagation loss in human tissue to be ∼1.5 dB per cm.

Despite that the fat tissue is lossy, we notice that the decay factor of the fat channel, presented in Figure 13b, is comparable to the decay factor of the free space channel, presented in Figure 10b. This observation is the motivation to compare the performance of the fat channel to similar waveguide structures, as we discuss in the next section.

### 4.2. Fat Channel as a Waveguide

The fat channel, as presented in the previous sections, consists of the skin, the fat, and the muscle layer. The structure of this channel is similar to the structure of parallel plate waveguides. The skin and muscle tissues can be seen as the top and bottom walls that guide the signal through the fat tissue. In this section, we experiment with our phantom model to compare the performance of the fat channel to waveguide structures.

**Figure 14:**
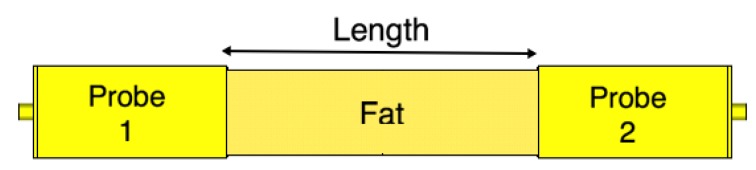
The fat channel model with the skin and muscle layers removed.

We remove the skin and the muscle tissue and compare the coupling coefficient between the two probes in the presence of the fat tissue only, as illustrated in Figure 14. In this case, the fat tissue is similar to a dielectric waveguide [36].

**Figure 15:**
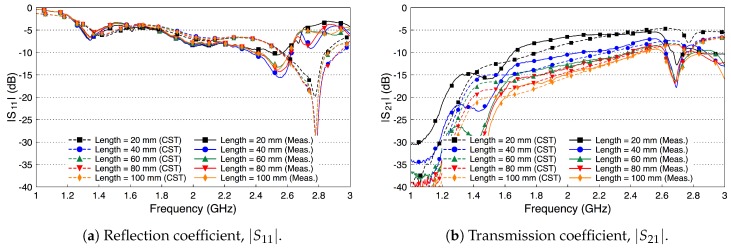
Measured and simulated scattering parameters of the probe-to-probe connection with the skin and the muscle layers removed and only the fat layer present.

Since the height of the fat tissue is finite, 25.36 mm, we expect the matching between the probe and the fat tissue to slightly deteriorate, since the probe is optimized to radiated to a fat tissue filling half the space. Figure 15a shows that this assumption indeed is true. Figure 15b shows that the coupling coefficient between the two probes decreases compared to the case when the skin and the muscle layers are present. The decrease in the coupling coefficient is caused by radiation of the signal into free space through the boundaries of the waveguide. We notice that the coupling coefficient increases as the frequency increases.

**Figure 16:**
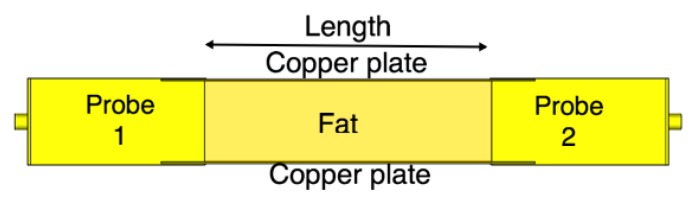
The channel model with parallel copper plates replacing the skin and the muscle layers.

**Figure 17:**
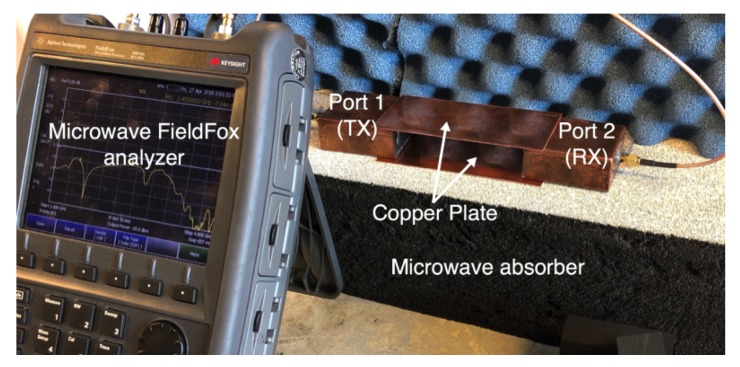
The experimental setup for the replacement of the skin and the muscle tissues with two parallel copper plates.

**Figure 18:**
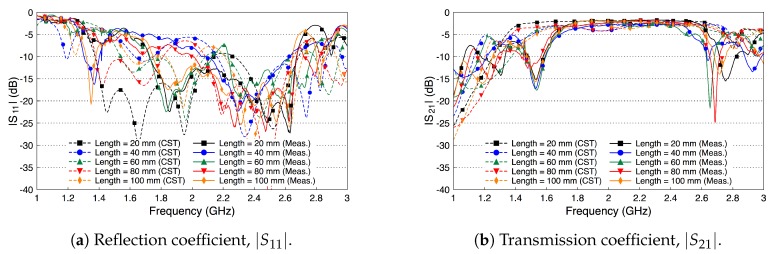
Measured and simulated scattering parameters of the fat channel when replacing the skin and the muscle tissues with two parallel copper plates.

We compare the performance of the phantom fat channel when the skin and the muscle tissues are replaced by two copper plates, as illustrated in Figure 16. This setup is similar to signal transmission in a parallel plate waveguides. The experimental setup is shown in Figure 17. The presence of the copper plates deteriorates the reflection coefficient of the probes, as illustrated in Figure 18a. The coupling coefficient between the probes is essentially flat over the R-band frequencies, as illustrated in Figure 18b. We notice that there is very little variation in the coupling coefficient when the channel length varies from 20 mm to 100 mm. Since the losses in our phantom are small, small variations in the coupling coefficient are expected. The replacement of the skin and muscle layers with copper plates results in lower path losses. A comparison between the coupling coefficients in Figure 12b, Figure 15b and Figure 18b shows that the three-layer tissue channel acts as a waveguide structure with path losses lower than the dielectric waveguide but higher than the copper plate waveguide.

**Figure 19:**
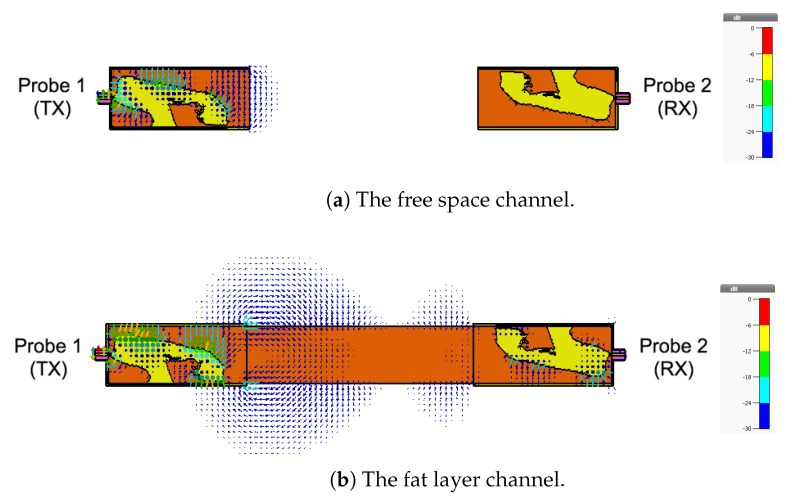

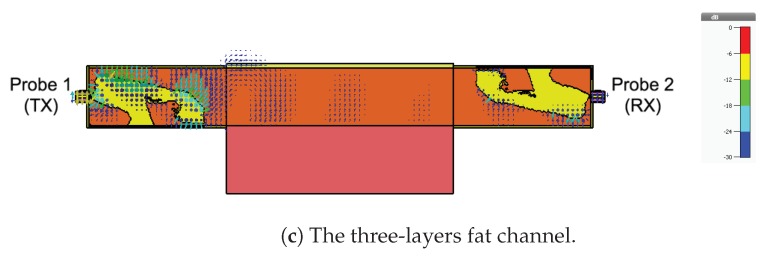
The simulated electric field distribution through the cross-section of the channel at 2.4 GHz for (**a**) free space; (**b**) the fat layer; and (**c**) the three-layer fat channel, with scalar color map.

As a final investigation, we use the electric field distribution to demonstrate the signal channeling between the two probes. We use the CST E-field monitor to study the electric field distribution at 2.4 GHz. Figure 19 shows a comparison of the electric field distribution between the two probes for the free space channel (Figure 8), the single-layer fat tissue (Figure 14), and the three-layer fat channel (Figure 2). Figure 19a shows that the E-field has high values only inside and at the aperture of the transmitting probe (the one to the left). The confinement of the E-field near the transmitting probe illustrates the mismatch between the probe and the free space. In Figure 19b, we notice that the presence of the fat layer allows the E-field to propagate out from the transmitting probe. Large portions of the field lines leak out of the fat layer to the surrounding free space. Although the fat layer improves the matching between the probe and the exterior domain, only small amounts of energy are guided to the second probe. For the three-layer fat channel, the E-field lines, shown in Figure 19c, show that the signal is launched from the probe and is confined between the skin and the muscle tissues. Moreover, the electric field lines are essentially normal to the muscle and skin layers inside the fat channel, which is similar to the orientation of the electric field of the Transverse Electric (TE) mode—that is, when the E-field component in the direction of propagation vanishes. It should be noted that some leakage from fat to air is prevalent along the vertical sides of the channel, but the orientation of the E-field is such that this leakage is much smaller than through the horizontal sides with the skin and muscle layers removed.

## 5. Conclusions

This work aims to assess the capability of the fat tissue as a microwave intra-body communication channel at R-band frequencies (1.7–2.6 GHz), which covers the Industrial, Scientific, and Medical (ISM) radio frequency 2.4 GHz. The communication medium is made up by the parallel plate transmission channel formed by the skin and the muscle layers together with the dielectric fat layer. A novel probe with a topology optimized planar antenna (TOPA) that provides a good signal match to the fat tissue has been designed and fabricated. We have conducted extensive studies in different setups and analyzed the propagation path loss in the three-layer tissue for the phantom and the ex-vivo porcine tissue. The measured transmission coefficient of the fat channel for phantom and ex-vivo environments were generally in agreement with simulation results. We estimate propagation path losses to ∼0.7 dB and ∼1.9 dB per cm for the phantom and ex-vivo, respectively. This difference can be mainly attributed to the conductivity of the fat tissue. The change in the fat conductivity between the phantom and the ex-vivo models is much more significant than the change in the skin and muscle conductivities as can be seen in Table 1.

The skin and the muscle tissues were found to perform as conducting layers, and a formation of these two different materials with high permittivity strongly affects the transmission signal through the fat tissue by confining the microwave signal in the fat tissue. The studies based on the transmission coefficient evaluations and the E-field distribution indicate that the presence of skin and muscle tissue enhances microwave propagation through the fat tissue. Overall, the presented results show the validity of our proposed technique for using the fat tissue as a new intra-body communication medium at R-band frequencies.

Microwave communication through fat tissue is a viable technique for a wireless implant to implant communication. Developing a reliable wireless fat channel based intra-body area network will help with gathering information from multiple implanted medical devices. It will enable future implant-based monitoring, controlled drug delivery, and sensor communication systems. Future studies will include the effects of blood vessels, take body movements into account, serving to represent a more realistic model of the working environment of the proposed communication technique. Moreover, safety issues including the use of bio-compatible materials for implant antenna development and computing the specific absorption rate (SAR) will be investigated. The results obtained from the current study pave the way for the development of a new wireless communication platform for implanted medical devices.

## Figures and Tables

**Table 1:** Dielectric properties of the phantom, ex-vivo, and human at 2.4 GHz.

Tissue	Parameter	Phantom	Ex-vivo	Human [28]
Skin	Conductivity	1.7 ± 0.2	1.4 ± 0.1	1.4 ± 0.1
	Relative permittivity	45 ± 4	31 ± 3	38 ± 4
	Loss tangent	0.28 ± 0.03	0.33 ± 0.03	0.28 ± 0.03
Fat	Conductivity	0.009 ± 0.001	0.12 ± 0.01	0.10 ± 0.01
	Relative permittivity	4.6 ± 0.4	3.5 ± 0.3	5.3 ± 0.5
	Loss tangent	0.015 ± 0.001	0.25 ± 0.02	0.15 ± 0.01
Muscle	Conductivity	1.8 ± 0.2	2.2 ± 0.2	1.7 ± 0.2
	Relative permittivity	52 ± 5	51 ± 5	53 ± 5
	Loss tangent	0.25 ± 0.02	0.32 ± 0.03	0.24 ± 0.02

## Abbreviations

WHO	World Health Organization
NCD	Noncommunicable Diseases
IMDs	Implantable Medical Devices
i-BAN	Intra-Body Area Network
RF	Radio Frequency
MICS	Medical Implant Communication Service
ISM	Industrial, Scientific, and Medical
CST	Computer Simulation Technology
FDTD	Finite Difference Time Domain
TOPA	Topology Optimized Planar Antenna
SMA	Sub-Miniature version A
TX	Transmitter
RX	Receiver
TE	Transverse Electric
E-Field	Electric Field
SAR	Specific Absorption Rate
